# Exploring the potential link between mRNA COVID-19 vaccinations and cancer: A case report with a review of haematopoietic malignancies with insights into pathogenic mechanisms

**DOI:** 10.18632/oncotarget.28827

**Published:** 2026-02-06

**Authors:** Patrizia Gentilini, Janci C. Lindsay, Nafuko Konishi, Masanori Fukushima, Panagis Polykretis

**Affiliations:** ^1^“Allineare Sanità e Salute” Foundation, Milano 20131, Italy; ^2^Independent Medical Scientific Commission (CMSi), Milano 20122, Italy; ^3^Toxicology and Molecular Biology, Toxicology Support Services, LLC., Sealy, TX 77474, USA; ^4^Osaka Metropolitan University School of Medicine, Osaka 545-0051, Japan; ^5^Learning Health Society Institute, Nagoya 450-0003, Japan

**Keywords:** COVID-19 genetic vaccines, adverse effects, cancer, lymphoblastic leukaemia, lymphoblastic lymphoma

## Abstract

This article investigates the potential association between modified mRNA (modRNA) COVID-19 vaccinations and the development of haematopoietic cancers. We present a case involving a healthy, young, athletic woman who developed acute lymphoblastic leukaemia (ALL) and lymphoblastic lymphoma (LBL) following her second dose of the Pfizer/BioNTech COVID-19 vaccine (Comirnaty^®^). This case is part of an expanding body of literature documenting similar occurrences after modRNA vaccinations, which we critically examine. Emerging evidence suggests that the biodistribution and persistence of modRNA, facilitated by lipid nanoparticles, can affect various tissues and organs, including the bone marrow and other blood-forming organs. Notably, modRNA vaccines exhibit a particular affinity for the bone marrow, potentially influencing the immune system at multiple levels and triggering both autoimmune disorders and neoplastic processes. In this article, we assess the risk of developing haematopoietic cancers post-modRNA vaccination based on current scientific literature and explore the reported potential genetic and molecular mechanisms involved in disease pathogenesis. By integrating clinical observations and current research, we aim to provide valuable insights into the potential carcinogenic outcomes associated with modRNA vaccination.

## INTRODUCTION

Acute lymphoblastic leukaemia (ALL)/lymphoblastic lymphoma (LBL) is a clonal haematopoietic stem cell disorder of B or T cell origin, and the World Health Organization 2017 Classification system categorizes these disease entities under “Precursor Lymphoid Neoplasms” [1]. Several authors have expressed concerns about the safety of modRNA vaccines for COVID-19 [2–11], which are technically pro-drug gene therapies encased in lipid nanoparticles (LNPs), rather than natural naked mRNA [6, 12]. The LNPs allow for unfettered access through most tissues and organs, including the brain and the bone marrow [13–15]. The mRNA is further modified (referred to as modRNA) by the substitution of all of the uridines for N1-methylpseudouridine (m1Ψ), in order to better stabilize the modRNA and also cloak it from the immune system [16].

In parallel to the roll-out of COVID-19 vaccines, an increase in excess mortality is being reported in several countries worldwide [17–21]. According to a recent study performed in Japan, the age-adjusted death rates for leukaemia, breast, pancreatic, and lip/oral/pharyngeal cancers increased significantly in 2022 after a large portion of the Japanese population had received the third dose of the modRNA vaccine, as compared to 2020, the first year of the pandemic, when no mass global genetic vaccinations were given [22]. Such study was subsequently retracted with a summary notice by the journal stating that: “*the correlation between mortality rates and vaccination status cannot be proven with the data presented*” [23], though without comprehensive evidence substantiating this claim. This underscores a critical literature gap: the absence of population studies verifying cancer incidence by vaccination status in order to estimate the true cancer incidence or mortality increases following COVID-19 vaccination. A study aimed to estimate the excess mortality in Germany for the years 2020–2022, showed that in 2020, there were approximately 4,000 excess deaths, while in 2021 and 2022, there were approximately 34,000 and 66,000 excess deaths, respectively [24]. A long-term population-wide cohort study in Pescara province (Italy), analysed nearly 300,000 residents over 30 months (June 2021 to December 2023) and investigated the relationship between COVID-19 vaccination and cancer hospitalizations [25]. The study found that vaccination was associated with a 23% increased risk of cancer hospitalization after receiving one or more doses, and a 9% increased risk after receiving three or more doses. Statistically significant increases in risk were observed for breast cancer (+54%), bladder cancer (+62%), and colorectal cancer (+35%) after at least one dose. Notably, while vaccinated individuals exhibited lower all-cause mortality, this could be attributed to “healthy vaccinee effect” [26] or to the “case-counting window bias” [27], rather than a direct protective effect. A large population-based retrospective cohort study from South Korea, involving over 8.4 million adults from 2021 to 2023, assessed the cancer risks within one year after COVID-19 vaccination [28]. The study found that vaccinated individuals had a 27% higher overall risk of developing cancer compared to those unvaccinated. Significant increases were noted for lung, prostate, thyroid, gastric, colorectal, and breast cancers. This elevated risk was consistent across vaccine platforms (including adenoviral vector, modRNA, and mixed schedules) with booster doses additionally linked to increased risks of gastric and pancreatic cancers. Increased cancer incidence was observed across all age groups and both sexes. Another study suggesting a possible link between spike protein expression and cancer progression after modRNA vaccination was recently published by Kuperwasser and El-Deiry, describing their review of 300 cases of cancers reported in the peer-reviewed literature following receipt of the COVID genetic vaccinations, and exploring possible mechanisms of oncogenesis [29]. Zhang and El-Diery had previously published on the ability of spike protein to suppress P53 activity in cancer cells potentially driving oncogenesis [30].

Despite over 30 years of research on mRNA technology as a gene therapy for treating various conditions (including genetic defects causing inborn errors of metabolism and cancers) this technology was first applied large scale globally as a genetic vaccine stimulating an immune response against a target viral antigen produced by self-cells rather than the virus. This novel approach was used to vaccinate healthy individuals against SARS-CoV-2 during the recent pandemic. Inoculation started early on, spanning all age groups, including vulnerable individuals and pregnant women, despite there being no long-term safety data, no randomized controlled trial (RCT) on pregnant women, and no data at all on genotoxicity or cancer [5, 6]. As relayed above, preliminary studies on carcinogenicity and genotoxicity were not conducted, and the randomized trial study of the Pfizer/BioNTech vaccine was prematurely halted after approximately 6 months, offering the placebo group the chance to vaccinate [31], thus losing any opportunity to understand the medium- to long-term repercussions, particularly concerning carcinogenesis. Although gene therapy has long been considered to pose an oncogenic risk, this is due to the phenomenon of insertional mutagenesis [6, 32–34].

Several studies report the occurrence of lymphadenopathies, often with suspicious characteristics in draining lymph nodes after vaccine administration, indicating significant stress on the immune system [35, 36]. A study on 951 patients who underwent PET-CT revealed that metabolic activity in axillary and supra-clavicular lymph nodes in 45.6% of vaccinated patients, especially after the second dose (53.9%) [36]. In 17 vaccinated patients (5.1%), “hot” lymph nodes reflected malignant lymph node disease, in 266 patients (80.1%), the “hot” lymph nodes were benign and vaccine-associated, while in 49 patients (14.8%), the nature of the lymph nodes was uncertain [36].

It is well-established that both natural and vaccine-derived spike proteins are toxic [37–40], but the latter is more persistent due to a double proline that confers greater stability. Additionally, the synthetic pseudouridines contained in the modRNA have shown mitochondrial toxicity in other applications, as warned of by the genetic vaccine developers Karikó and Sahin [41]. Furthermore, it has been demonstrated that this modification can increase the likelihood of +1 ribosomal frameshifting during translation, resulting in the production of multiple peptide products with unexplored effects [42]. This obviously poses serious safety concerns as only a single antigen was supposed to be encoded by the modRNA, not many undefined peptides with unknown antigenic and autoimmune potential. The encapsulation of the modRNA in LNPs not only allows for systemic diffusion [43] but also exhibits intrinsic cytotoxicity, raising significant concerns [44, 45]. Furthermore, the LNPs display a wide distribution beyond the injection site, involving multiple tissues, including the bone marrow [5, 13–15], which affects the haematopoietic process. Specifically, the Therapeutic Goods Administration (TGA) documents related to the Pfizer/BioNTech vaccine nonclinical evaluation reveal an accumulation of modRNA-containing nanoparticles in various tissues. Notably, the vaccine begins to accumulate rapidly, particularly in the bone marrow. Between 30 minutes and 48 hours following intramuscular injection, the concentration of radioactively labelled nanoparticles in the femoral bone marrow of rats increased by 7.9-fold [15].

Recent case reports and mechanistic insights suggest that modRNA COVID-19 vaccines may, in some instances, contribute to the development or rapid progression of cutaneous lymphomas, such as mycosis fungoides, through an oncomodulatory rather than direct oncogenic effect [46, 47]. The proposed mechanism involves vaccine-induced activation of innate immune pathways, including Toll-like receptors (TLRs), which stimulate NF-κB signalling and induce proinflammatory cytokine production (e.g., IL-6). This cytokine milieu subsequently activates the STAT3 pathway, both of which are known to promote malignant T-cell proliferation, survival, and resistance to apoptosis in cutaneous T-cell lymphoma. Additionally, repeated vaccination may exacerbate CD30 overexpression and T-cell exhaustion, impairing immune surveillance and enabling tumour growth in predisposed individuals [46, 47]. However, it must be noted that specific studies detailing how the modRNA interacts with the immune system or bone marrow at the molecular level are currently lacking. Our study aims to draw attention specifically to this gap in the literature and to provide supporting evidence for plausible molecular and immunological mechanisms that might underlie these rare but concerning observations.

While meta-analyses and systematic reviews predominantly indicate that COVID-19 modRNA vaccines are safe and provide clinical benefit to cancer patients [48, 49], including those with haematological malignancies [50, 51], a recent retrospective cohort study presents contrasting results regarding the effects of repeated COVID-19 booster vaccinations on patients with pancreatic cancer [52]. In particular, this study reported that repeated booster doses, were associated with poorer overall survival, correlating with increased levels of the immunosuppressive subtype immunoglobulin G4 (IgG4), which was spike-specific [52]. This same study supports a potential immuno-modulatory mechanism for IgG4 within the tumour microenvironment, namely that elevated IgG4 levels and increased Foxp3-positive regulatory T-cells may impair the normal anti-tumour immunity of T cells. Previous studies have shown that IgG4 can promote immune evasion by blocking other immunoglobulin receptor functions and reducing CD8+ T-cell infiltration of tumours [53, 54]. This highlights the varied complexity of modRNA and other genetic vaccine potential impacts on cancer prognosis through immune modulation following constant antigen presentation and tolerance leading to immunoglobulin class switching. This point to the necessity for continued research into both the acute and long-term impacts of repeated genetic vaccinations in diverse cancer populations. We do acknowledge that these studies were conducted on patients already diagnosed with cancer, which limits their ability to provide definitive evidence for interpreting a potential causal link between modRNA vaccination and the development or progression of malignancies. Therefore, these findings should be interpreted with caution and highlight the need for further prospective and molecular studies specifically designed to evaluate these possible connections.

## CASE REPORT

The case involves a 38-year-old woman who received the second dose of Comirnaty^®^ in July 2021. Before the symptoms appeared, she maintained a healthy lifestyle and participated in athletic activities like pole dancing and callisthenics. There is no significant family or pathophysiological history. Previous laboratory assessments, conducted concurrently with her athletic activities (in 2016, 2017, 2019 - most recent on April 13, 2021), did not yield any noteworthy findings.

### Recent medical history

In April 2021, during a routine check-up for sports practice, an occasional finding of modest leukopenia was observed: white blood cells (WBC) 2450/μL (normal range: 5000–10000/μL), neutrophils 650/μL (normal range: 1900–8000/μL), equivalent to 26.5% (normal range: 40–74%), lymphocytes 68.2%, no atypical forms, normal Hb (12.7 g/dl), and platelets (195,000/mm³). The physician did not prioritize the issue, and no additional follow-up assessments were advised. The first dose of Comirnaty^®^, administered on June 20, 2021, did not induce notable symptoms. On the morning of July 20, 2021, the day after the administration of the second dose of Comirnaty^®^ (both administrations occurred at public facilities), the patient experienced significant discomfort. She woke up with a locked neck and jaw, tinnitus, nausea, diffuse pain, low-grade fever, headache, and sweating. Symptoms worsened in the following days, accompanied by insomnia, hypersensitivity to temperature changes, and noise. The patient consulted her primary care physician and took ketoprofen lysine salt 80 mg (OKI^®^), and paracetamol 500 mg (Tachipirina^®^), resulting in only a mild and transient reduction in symptoms. Due to persistent symptoms, on August 6, 2021, haematological tests were performed, revealing altered blood counts with neutropenia and lymphocytosis (refer to the discussion for references): WBC 5230/μL (normal range: 5000–10000/μL), neutrophils 1400/μL (normal range: 1900–8000/μL), equivalent to 26.8% (normal range: 40–74%), lymphocytes 3390/μL, equivalent to 64.8% (normal range: 19–48%), elevated erythrocyte sedimentation rate (ESR) at 59 mm/hour (normal female <50 years-old range: ≤20 mm/hour), transferrin 233 mg/dL (normal female range: 250–380 mg/dL). Haemoglobin, platelets, liver, and kidney function indices were within normal ranges. As the subjective symptoms continued to be increasingly disabling, further examinations were conducted: (i) On September 8, 2021 a laboratory check revealed mild anaemia (Hb 10.8 g/dL, normal range: 12.3–15.3 g/dL), mean cell volume (MCV) 103.6 fL (normal range: 80–100 fL), neutrophils 990/μL (normal range: 1900–8000/μL), equivalent to 22.9% (normal range: 40–74%), increased lymphocytosis (lymphocytes 70.4%, normal range: 19–48%), and ESR 66 mm/hour (normal female <50 years-old range: ≤20 mm/hour). Other parameters, including C-reactive protein, complement factors, rheumatoid factor, thyroid-related antibodies, were within normal limits; on (ii) October 1, 2021 the laboratory analyses confirmed anaemia (Hb: 10.4 g/dL, normal range: 12.3–15.3 g/dL), neutrophils 1370/μL (normal range: 1900–8000/μL), equivalent to 29% (normal range: 40–74%), persistent lymphocytosis (lymphocytes 65.8%, normal range: 19–48%), mean cell volume (MCV) 103.4 fL (normal range: 80–100 fL) and an elevated ESR 96 mm/hour (normal female <50 years-old range: ≤20 mm/hour). Homocysteine, creatine kinase, and C-reactive protein were normal. Serological tests for hepatitis, rubella, Epstein-Barr, Cytomegalovirus, Treponema, Toxoplasma, as well as autoantibodies (ANA, ANCA, ENA, ADNA, and anti-citrulline) were all negative; on (iii) October 16, 2021 the ESR was 118 mm/hour (normal female <50 years-old range: ≤20 mm/hour). The ESR increased progressively from August 6 to October 16 and was as follows: 59-66-96-118 mm/hour. A rheumatological examination on October 22, 2021, suggested post-vaccination inflammation following the second dose of Comirnaty^®^ (July 19, 2021), with symptoms including arthromyalgia, headache, low-grade fever, night sweats, and an ESR of 118 mm/hour. The patient was diagnosed with polymyalgia rheumatica (PMR)/vasculitis of large vessels post-vaccination. She was recommended to undergo a PET scan with a big vessel wall uptake analysis, and steroid therapy afterward. PET scan on November 15, 2021, revealed intense uptake in the medullary component of the entire axial and appendicular skeleton and diffuse increased uptake in the spleen. Suspecting lymphoproliferative pathology, urgent haematological consultation was initiated, leading to a haematological examination, bone marrow aspiration, and biopsy on December 1, 2021.

### Clinical examination and diagnosis

Clinical examination revealed no superficial lymphadenopathy, and the abdomen and chest were normal. The patient experienced significant diffuse pain and intense sweating from early October, persisting until the beginning of chemotherapy, with a brief interval following corticosteroid therapy. Blood analysis on November 29 showed Hb 9.1 g/dL, WBC 4030/mm³, neutrophils 1810/mm³, lymphocytes 2180/mm³, circulating atypical lymphoid elements, rare immature myeloid elements, C-reactive protein 2.18 mg/dL (normal range: 0.3–1.0 mg/dL), beta-2 microglobulin (B2M) level of 2.7 mg/dL (normal range: <0.2 mg/dL). Bone marrow aspirate and biopsy revealed near-total replacement of haematopoietic components by a massive and widespread infiltrate of blast-like elements (approximately 95% of nucleated cells), with irregular or cleaved nuclei characterized by the following immunophenotypic profile: TdT(+), CD34(+), CD79a(+), PAX5(+), CD20(−/+), CD10(+), MYC(−/+), CD3(−), CD5(−), Cyclin D1(−), CD23(−), pg53(−), MPO(−), residual haematopoietic component represented by scattered erythroblasts and rare dystrophic megakaryocytes. The immunophenotypic profile indicated a precursor B-lymphoid neoplasm, specifically B-lymphoblastic leukaemia/lymphoma (according to the WHO classification). The patient initiated the prescribed chemotherapy protocol, achieved complete remission, and is currently undergoing maintenance therapy. There are no specific “pre-leukaemic” alterations of ALL, and it cannot be ruled out that in April 2021, some dysfunction of haematopoiesis was already underway. Likewise, it cannot be certain - if this were the case - that the administration of Cominarty^®^ did not only accelerate, but also contributed to the definitive malignant transformation in light of the profound interactions on the immune system induced by the modRNA products.

### Follow-up and subsequent therapeutic interventions

In October 2024, after 23 cycles, the patient discontinued maintenance therapy - which included 12 diagnostic/therapeutic lumbar punctures, all negative for relapse - due to myalgia, fever with repeated episodes of bacteraemia, and thrombosis. On October 22, 2024, approximately three years after disease onset, complete remission (including molecular remission) was still confirmed. However, in January 2025, the patient developed difficulty walking, headache, and neck pain, leading to a diagnosis of central nervous system relapse. Cerebrospinal fluid analysis showed 1,082 white blood cells/mm³, of which 80% were lymphoblastic cells expressing CD19(+), CD34(+), CD10(+/– 30%), and CD45(–). The patient began systemic therapy with high-dose cytarabine and methotrexate, along with therapeutic lumbar punctures, and was scheduled for an allogeneic stem cell transplant from an unrelated donor on April 16, 2025, following a conditioning regimen consisting of fludarabine 40 mg/m² and Total Body Irradiation (TBI) 12 Gy. The hospitalization was complicated by febrile neutropenia, diarrhoea, stomatitis, reactivation of herpes simplex virus type 2, and thrombosis at the central venous catheter site. Approximately two weeks after the transplant, leukocyte and platelet engraftment were achieved, and the patient was discharged on May 4, 2025. Currently, the patient’s condition is gradually improving. She continues with follow-up visits and immunosuppressive therapy with cyclosporine and is awaiting a comprehensive reassessment. Table 1 presents a schematic summary of the events described in this case report.

**Table 1:** Schematic timeline of clinical events, laboratory findings, diagnoses, and therapeutic interventions

Date	Event and Findings
April 2021	Mild leukopenia (WBC 2.450/u.L) at routine check-up
June 20, 2021	First dose Comimaty^*^; no notable symptoms
July 20, 2021	Second dose Comimaty^®^; acute symptom onset (fever, neck/jaw rigidity, pain)
Aug-Oct 2021	Neutropenia, lymphocytosis, rising ESR (59→118 mm/h); PMR/vasculitis diagnosis after rheumatology exam
Nov 15, 2021	PET scan: diffuse bone marrow/splenic uptake
Dec 1, 2021	Bone marrow biopsy: B-lymphoblastic leukaemia/lymphoma diagnosis
2022–2024	Chemotherapy and maintenance therapy; complete remission
Oct 2024	Maintenance therapy stopped due to complications; remission confirmed
Jan 2025	CNS relapse confirmed by CSF; 80% lymphoblasts
Apr-May 2025	Allogeneic stem cell transplant and discharge
Present	Gradual recovery, ongoing immunosuppressive therapy

## DISCUSSION

### Haematological malignancies and lymphoproliferative disorders following COVID-19 vaccination

Several papers, mostly case reports, describe malignancies that developed in close temporal relationship with modRNA COVID-19 vaccinations. A total of 30 studies were identified, with 28 focusing on haemato-lymphoproliferative disorders. Among the case reports, there are 9 cases of B-cell lymphoproliferative disorders, 13 involving the T-cell line, 6 affecting the myeloid line, and 2 cases related to the onset of solid tumours. A summary is presented in Tables 2–4, detailing cases involving the lymphoid series categorized by B and T phenotypes, and the myeloid series, respectively.

**Table 2:** Lymphoproliferative disorders following COVID-19 vaccination with a B-phenotype

Case No.	Sex/Age (ref.)	Time elapsed from vaccination to onset of symptoms	Histology	Vaccine type	Site
1	F/58 [55]	1 week	DLBCL	Pfizer/BioNTech (2nd dose)	Left cervical area
2	F/80 [56]	1 day	MZL	Pfizer/BioNTech (1st dose)	Right temporal lobe
3	M/51 [57]	7 days	DLBCL	Astra Zeneca (1st dose)	Mediastinum
4	M/67 [58]	2 weeks	DLBCL	Pfizer/BioNTech (2nd dose)	Axilla
5	F/80 [58]	2 days	DLBCL	Pfizer/BioNTech (2nd dose)	Axilla
6	F/49 [59]	2 days	B-ALL	Pfizer/BioNTech (dose n.s.)	Bone marrow
7	F/47^*^ [59]	Few days	B-ALL	Pfizer/BioNTech (dose n.s.)	Bone marrow
8	F/43 [60]	Few days	B-ALL	Moderna (dose n.s.)	Bone marrow
9	F/61 [61]	Few weeks	IVLBCL	Pfizer/BioNTech (2nd dose)	Multi-organ blood vessels

Abbreviations: DLBCL: Diffuse large B-cell lymphoma; MZL: Marginal zone B-cell lymphoma; B-ALL: Acute lymphoblastic leukaemia B; IVLBCL: Intravascular large B-cell lymphoma. ^*^Patient in remission for two years after treatment for non-Hodgkin’s lymphoma.

**Table 3:** Lymphoproliferative disorders following COVID-19 vaccination with a T-phenotype

Case No.	Sex/Age (ref.)	Time elapsed from vaccination to onset of symptoms	Histology	Vaccine type	Site
1	M/53 [55]	3 days	ENKTCL	Pfizer/BioNTech (1st dose)	Oral cavity
2	M/66 [62]	1 week	AITL	Pfizer/BioNTech (2nd dose)	Lymph nodes
3	M/73 [63]	3 months	ENKL	Pfizer/BioNTech (2nd dose)	Injection site
4	F/28 [64]	3 days	SPTCL	Janssen Pharmaceuticals	Injection site
5	M/45 [65]	3 days	SPTCL	Moderna (dose n.s.)	Periumbilical region
6	M/76 [66]	10 days	ALCL	Moderna (3rd dose)	Injection site
7	M/60 [67]	4 weeks	CTCL	Astra Zeneca (dose n.s.)	Occipital area
8	F/73 [67]	10 days	CTCL	Astra Zeneca (dose n.s.)	Skin
9	M/66 [68]	10 days	ALCL	Pfizer/BioNTech (3nd dose)	Cervical an axillary lymph nodes
10	M/55 [69]	2 days	T-ALL NK	mRNA (brand & dose n.s.)	Neck lymph node and bone marrow
11	M/79 [70]	3 days	CTCL	Moderna (3nd dose)	Injection site
12	F/79 [46]	1 month	CTCL	Pfizer/BioNTech (2nd dose)	Skin and regional lymph nodes
13	F/56 [71]	2 days	CTCL	Pfizer/BioNTech (1st dose); Moderna (2nd dose)	Skin

Abbreviations: ENKTCL: Extranodal malignant non-Hodgkin lymphoma with T/NK cells; AITL: Angioimmunoblastic T-cell lymphoma; ENKL: Extranodal NK/T-cell lymphoma, nasal type; SPTCL: Panniculitis-like T-cell lymphoma; ALCL: Anaplastic large cell lymphoma; CTCL: Cutaneous T-cell lymphoma; T-ALL NK: T Cell lymphoblastic leukaemia with NK phenotype.

**Table 4:** Myeloproliferative disorders following modRNA COVID-19 vaccination

Case No.	Sex/Age (ref.)	Time elapsed from vaccination to onset of symptoms	Histology	Vaccine type	Site
1	F/67 [59]	2 months	AML^*^	Pfizer/BioNTech	Bone marrow
2	M/60 [72]	1 month	AML	Pfizer/BioNTech (4th dose)	Bone marrow
3	M/61 [72]	1 month	AML	Pfizer/BioNTech (3rd dose)	Bone marrow
4	M/72 [72]	5 weeks	AML	Pfizer/BioNTech (5th dose)	Bone marrow
5	F/28 [72]	4 weeks	AML	Pfizer/BioNTech (2nd dose)	Bone marrow
6	F/74 [66]	4 days	CMML	Janssen Pharmaceuticals	Bone marrow

Abbreviations: AML: Acute myeloid leukaemia; CMML: Chronic myelomonocytic leukaemia. ^*^Return of AML into remission after allogeneic transplant 14 years earlier.

In the overwhelming majority of cases, there is a *de novo* onset of proliferative disorders affecting the lymphoid lineage, encompassing both B and T phenotypes. The Pfizer/BioNTech vaccine appears to be the most implicated (16 cases). The onset of symptoms following vaccine inoculation has generally been very brief, even within a few days, as seen for instance in the cases reported by Kreher et al., Ukishima et al., and Panou et al. [64, 65, 67]. In one case, acute lymphoblastic leukaemia occurred in a 47-year-old woman who had been in remission for two years from a B-cell lymphoma [59]. Two cases of T-cell lymphomas exhibited a recurrence of previously well-controlled conditions (mucosis fungoides and lymphomatoid papulosis) [67]. Notably, in lymphoma cases, four cases showed onset at the inoculation site [63, 64, 66, 70], and three cases manifested in draining lymph nodes (axillary and lateral cervical) [55, 58, 68]. An interesting case involves angioimmunoblastic T-cell lymphoma, where rapid progression was observed after the booster dose [68]. The patient received two doses of Comirnaty^®^ around March-April 2021, approximately 5 and 6 months before lymphoma onset. On September 8, 2021, a baseline PET/CT revealed hypermetabolic lymph nodes mainly in the supra-clavicular, cervical, and left axillary regions, as well as restricted gastro-intestinal hypermetabolic lesions consistent with lymphoma involvement. Following a booster dose administered on September 22, 2021, a follow-up PET/CT on September 30 showed a dramatic increase in both nodal and gastro-intestinal hypermetabolic lesions, with notable asymmetrical metabolic progression in the cervical, supra-clavicular, and axillary areas (particularly pronounced on the side of the booster injection). This unusually rapid and localized disease progression suggests a possible link between immune activation by the booster and lymphoma acceleration.

Regarding solid tumours that developed soon after receiving the modRNA COVID-19 vaccination, two cases were reported: one involved a 64-year-old woman who had a significant history of previously excised cutaneous melanoma reoccurring to the breast [73], and the other involved an aggressive sarcoma that developed at the injection site shortly after the second dose of Moderna [74].

Regarding the pre-vaccination neutropenia observed this case report in April 2021, although rare pancytopenic prodromes preceding overt ALL have been reported in ~1.3–2.2% of paediatric cases, characterized by severe pancytopenia and abnormal lymphoid cells in bone marrow aspirates [75], such features are not typical of ALL, which is a rapidly progressive malignancy characterized by the sudden accumulation of lymphoblasts in the marrow and blood [76]. The patient’s isolated mild leukopenia (WBC 2,450/μL, neutrophils 650/μL, normal Hb/platelets, no atypical forms), detected during routine wellness screening for sports practice, more likely reflects a benign transient neutropenia from viral or other non-malignant causes rather than smouldering leukemia. Notably, the complete blood count one month post-second vaccine dose showed absolute neutrophils more than doubled to 1400/μL, with normal WBC, Hb, and platelets. A precursor ALL process would be unlikely to show post-vaccination neutrophil increase alongside no evidence of anaemia or thrombocytopenia.

### Potential carcinogenic mechanisms induced by COVID-19 modRNA vaccines

Several mechanisms have been proposed by which the current modRNA COVID-19 vaccines may exert a carcinogenic effect, inducing both *de novo* tumour formation and the recurrence of neoplastic diseases in remission. It is important to note that, as genetic therapeutic products (GTPs), modRNA vaccines, have been associated with a potential risk of inducing cancer and haematological disorders [6, 77]. The main alterations induced by modRNA COVID-19 vaccines reported in literature, that may have an oncogenic outcome, are listed below:

- The alteration of the inhibitory immune checkpoint mediated by the programmed cell death protein 1 (PD-1, CD279), which is primarily found on T-cells, mature B-cells, and other immune cells. The overexpression of the programmed death-ligand 1 (PD-L1), observed in vaccinated individuals, leads to T-cell immunosuppression, impairing cancer surveillance [78].
- The interaction between the S2 subunit of the spike protein and the oncosuppressor proteins p53, BRCA1, and BRCA2, which regulate downstream genes in response to numerous cellular stresses and play a crucial role in preventing cancer [30, 79].
- The impairment in type I interferon (IFN) signalling, which plays essential roles in inflammation, immunomodulation, tumour cell recognition, and T-cell responses [80]. Differential gene expression analysis of peripheral dendritic cells revealed dramatic up-regulation of type I and type II IFNs in COVID-19 patients, but not in vaccinees. All this supports the possibility that COVID-19 genetic vaccines actively suppress the production of type I IFN, which plays a fundamental role in the immune reaction in response to multiple stressors, especially viral infections and tumours. In the presence of viral infection, the production of type I IFN drastically increases, and IFN-α released from lymph nodes induces B-cells to differentiate into plasma blasts, which then further differentiate into antibody-secreting plasma cells under the action of IL6. As for the anti-tumour action of IFNs, its mechanisms of action include both direct and indirect effects. Direct effects include cell cycle arrest, induction of cell differentiation, initiation of apoptosis, and activation of natural killer and CD8^+^ T-cells. The indirect anti-tumour effects are mainly due to the activation of transcription factors, which improve the expression of at least 150 genes also involved in apoptosis.
- Increased Transforming Growth Factor Beta (TGF-β) Production. The interaction between the SARS-CoV-2 spike protein and the angiotensin-converting enzyme 2 (ACE2) induces TGF-β release by cells such as alveolar and tissue macrophages, lung epithelial cells, endothelial cells, and B lymphocytes, promoting epithelial-mesenchymal transition (EMT) [40, 81]. This process could explain the particular rapidity of onset and evolution of tumour forms arising following the administration of the COVID-19 genetic vaccines. In fact, the TGF-β is a growth factor capable of inducing in already differentiated cells a “regression” towards the mesenchymal state (a state typical of the early stages of embryonic life), with the ability to metastasize and greater biological aggressiveness.
- The presence of LNP-encapsulated DNA contamination originating from residual plasmid DNA used during the manufacturing process of the Pfizer/BioNTech and Moderna modRNA vaccines [82–85]. The residual DNA detected in the modRNA genetic vaccines is high in copy number and contains elements such as: functional promoters, open reading frames (ORFs), origins of replication, and nuclear targeting sequences. In the case of the Pfizer/BioNTech genetic vaccine, such plasmids have been engineered with a mammalian SV40 promoter-enhancer-ori from the oncogenic virus Simian Virus 40 (SV40) along with a nuclear targeting sequence (NTS) [82–85]. In fact, Health Canada requested Pfizer to provide data on the size distribution of DNA fragments in its COVID-19 vaccines, specifically due to concerns about the potential for these fragments to integrate into human genomes, which could pose safety risks [86]. This request came after Health Canada discovered that Pfizer had withheld information about certain DNA sequences, including residual plasmid DNA and the undisclosed SV40 enhancer element, present in the genetic vaccines. This human-compatible promoter is not required for the expression of these plasmids in the *E. coli* bacterial expression system, and its presence is highly questionable, as it poses a significant oncogenic risk that is not needed for the plasmid’s stated purpose [87]. Although modRNA vaccines are not classified as DNA-based, the FDA’s guidance for plasmid DNA vaccines applies to the contaminating plasmids used in their production, which carry eukaryotic promoters and enhancers posing similar risks of insertional mutagenesis. FDA advises the following: “*Plasmid biodistribution, persistence and integration studies were initially recommended to examine whether subjects in DNA vaccine trials were at heightened risk from the long-term expression of the encoded antigen, either at the site of injection or an ectopic site, and/or plasmid integration. Theoretical concerns regarding DNA integration include the risk of tumorigenisis if insertion reduces the activity of a tumour suppressor or increases the activity of an oncogene. In addition, DNA integration may result in chromosomal instability through the induction of chromosomal breaks or rearrangements.*” [88]. In this context, it is essential to recall the insightful words of virologist Dr. Reinhard Kurth, who emphasized the importance of weighing risks against benefits, particularly noting that the risk/benefit ratio in gene therapy differs significantly from that in DNA vaccination, where vaccinees are generally healthy individuals rather than seriously ill patients: “*When discussing risks, one cannot overlook potential benefits. Obviously, the risk/benefit ratio in gene therapy is very different from that in DNA vaccination. In the former, patients are normally treated who suffer from very serious diseases and who possess a very poor prognosis. In contrast, vaccinees are usually young and healthy. Thus, the higher relative risk in nucleic acid vaccination (because vaccinees are not patients) is an important aspect in the ongoing discussions about safety*” [89]. Building on this perspective, the WHO Expert Committee on Biological Standardization (Geneva 1998) further clarifies the need for careful preclinical safety evaluation: “*After injection of DNA into an animal, only a small proportion of the DNA molecules enter cells, and of those that do, only a fraction is likely to enter the nucleus. The probability of an extraneous DNA molecule being integrated into a chromosome is very low. When consideration is given to the probability of insertional mutation occurring at a growth-regulatory gene, and to the multi-step process of oncogenesis, the risk of insertional mutagenesis is seen to be exceedingly low. This argument is based upon the known low frequency of DNA insertions* in vitro *in replicating cells specifically treated to enhance DNA uptake. There is relatively little data on the frequency of DNA insertion in tissue cells* in vivo, *and none to suggest that it may be higher than that observed* in vitro. *Nevertheless, an important aspect of the preclinical safety testing of a DNA vaccine is investigation of the potential of* in vivo *integration of plasmid DNA into the vaccinated subject’s chromosomes, especially since such vaccines are likely to contain strong eukaryotic- or viral-transcription promoters*” [87]. This cautious approach is echoed in the EMA Guideline on plasmid DNA vaccines for veterinary use, which expands on the potential risks of chromosomal integration and the necessity for sensitive integration studies: “*The plasmid DNA which is internalised by the cells of the vaccinated animal may integrate into the chromosomes of the vaccinated animal and disrupt the normal replicative state of that cell, causing uncontrolled cell division and oncogenesis… After the injection of DNA into an animal, a small proportion of the DNA molecules enters cells. The probability of any DNA molecule integrating into the chromosome is low and given that oncogenesis is a multi-factorial event, the risk of insertional mutagenesis is exceedingly low. Integration studies, where relevant, should be undertaken with the finished product and the percentage of supercoiled plasmid used should be stated. So far, the integration of plasmid DNA into chromosomal DNA of a vaccinated animal has not been observed (EFSA, EFSA Journal 2017). However, integration (e.g., into the muscle cells surrounding the vaccination site or into germ line cells in the gonads) cannot be discounted. The current testing methods are not sufficiently sensitive to routinely detect actual integration that may be orders of magnitude below the limits of detection of the methods. Therefore, each product should be assessed on a case-by-case basis, taking into consideration the specific limits of detection, the route of administration, the target tissue, the amount of plasmid administered, and the age of the vaccinated animal. The information should be compiled in a risk assessment.*” which continues at page 10: “*If plasmid DNA is detected, suitably sensitive methods should be used to investigate possible integration of plasmid DNA into the host genome. If integration is detected or suspected, and a risk of oncogenicity due to the life expectancy of target animals is identified, a test for oncogenicity in a susceptible laboratory animal system could be carried out. Alternatively, the incidence of tumours in the target species, particularly at the site of injection and in the target tissue, could be recorded at the end of pivotal target animal safety and relevant efficacy studies (e.g., duration of immunity)*” [90]. To the best of the authors’ knowledge, none of the recommended evaluations addressing the potential for *in vivo* integration of plasmid DNA have been conducted for COVID-19 modRNA vaccines, and adequate preclinical safety testing in this regard remains lacking. On the contrary, some independent studies report that the amount of the contaminating plasmids is far above the regulated limits for naked DNA contamination on vaccines [82–84]. This has been confirmed even by a study conducted at the FDA White Oak Campus which found alarmingly high levels of DNA contamination in Pfizer’s modRNA COVID-19 vaccine, with estimated amounts of residual DNA in one human dose exceeding safety limits by 6 to 470 times [85]. It should also be specified that, for insertional mutagenesis risk, alongside mere total DNA mass, assessments should consider the number of molecules, as more molecules increase the probability that a molecule’s ends match a potential insertion site. Insertional mutagenesis frequently leads to cancer, and gene therapy has long been recognized to carry an oncogenic risk, as acknowledged by the FDA in their guidance on plasmid DNA vaccines [91], and supported by the studies previously cited [32–34]. In fact, as relayed earlier, the SV40 virus is a known oncogenic virus when intact [92, 93]. There is also the additional potential for the modRNA to be reverse transcribed to DNA through the reverse transcriptase activity of LINE-1, as previously demonstrated by Aldén et al. [94], especially in tissues such as the testes and ovaries, as well as the bone marrow that are rich in this transcription factor [83, 94]. Recently, Prof. Shigetoshi Sano reported a case of rapid breast cancer skin metastasis following the 6th dose of Pfizer/BioNTech modRNA vaccine [95]. The metastatic cancer cells were found to express the vaccine-derived spike protein, but not the viral nucleocapsid protein, suggesting a possible link between spike protein expression and cancer progression after vaccination. This novel finding underscores the urgent need for further research into the oncogenic potential of mRNA vaccines and their role in cancer progression.
- The role of the immunoglobulin subtype IgG4 in immunomodulation contributing to cancer endpoints including immunosuppression and immune evasion. Wang et al. found that IgG4-containing B lymphocytes and IgG4 concentration were significantly increased in cancer tissues, as well as in the serum of patients with cancer [54]. Both were positively correlated with worse prognoses and increased cancer malignancy. Previous studies have reported that IgG4 is locally produced in melanoma, playing an important role in evading immune system control and promoting tumour progression [53, 96]. The increased production of IgG4 occurs with prolonged and repeated exposures to singular antigens and their interaction with antibodies of the IgG and IgE classes through their Fc domains [97]. IgG4 is in fact endowed with a dual role, as it can suppress or stop inflammation by competing with inflammatory IgE for binding to the antigen, in the case of allergies and infections from helminths and filarial parasites or, on the contrary, IgG4 can lead to serious autoimmune diseases [98] and cancer, playing an essential role in the “immune evasion” of cancer cells [99]. Recent studies indicate that repeated modRNA vaccinations against COVID-19 shifts the antibody response towards the IgG4 subclass with a decrease in FcγR-dependent effector activity and an increased COVID-19 infection fatality rate [100–102]. In cohorts of healthy healthcare workers, it was demonstrated that several months after the second dose, the SARS-CoV-2-specific antibodies were increasingly composed of immunosuppressive IgG4, which were further increased by a third modRNA vaccination and/or by subsequent infections of SARS-CoV-2 viral variants [101]. IgG4 antibodies, among all spike-specific IgG antibodies, increased on average from 0.04% shortly after the second vaccination to almost 20% after the third vaccination [102]. Spike protein/galectin-3 molecular mimicry may facilitate recruitment of vaccine-induced IgG4 to the tumour microenvironment [103]. Once localized there, IgG4 promotes cancer progression through specific immunosuppressive mechanisms: binding anti-tumour IgG1 antibodies to block effector cell function, engaging inhibitory FcγRIIB receptors on innate immune cells, and creating oncogenic microenvironments through epitope targeting [103]. Moreover, a review of 10 studies on patients with IgG4-related disease (IgG4-RD), which features excess IgG4, revealed elevated rates of several cancers, particularly pancreatic cancer and lymphoma [99]. Perugino et al. identified IgG4 anti-galectin-3 autoantibodies in 28% of IgG4-RD patients, correlating with elevated IgG4/IgE levels and consistent with B cell-driven class switching mechanisms potentially triggered by galectin-3 [104]. Galectin-3 shares near-identical homology with the spike protein’s N-terminal domain, potentially driving IgG4 switching via molecular mimicry [103–105]. Mechanistically, tumour cells expressing vaccine-derived spike protein recruit spike-specific IgG4 (induced by repeated mRNA vaccination/galectin-3 mimicry) to the tumour microenvironment, promoting cancer progression by: (a) binding anti-tumour IgG1 to block effectors, (b) engaging inhibitory FcγRIIB receptors, and (c) creating oncogenic microenvironments [103]. Thus, repeated mRNA vaccines may drive cancer progression via spike-specific IgG4 recruitment and immunosuppression.
- The incorporation of m1Ψ into the modRNA of the genetic vaccines causes ribosomal frame-shifting during translation, which can lead to the production of numerous peptide products that are expressed differentially in each individual, as well as may cause lethal mitochondrial toxicity as was discussed by the developers of the technology, Karikò and Sahin in their 2014 review [41, 42]. Given that these unidentified peptides may have unknown antigenic and auto-immune potential, they pose a serious risk of carcinogenesis and therefore require further investigation.
- A recent hypothesis paper proposes that mRNA vaccines’ LPNs, via hepatic tropism, may transiently dysregulate liver metabolism in susceptible individuals, potentially promoting leukemogenesis through five mechanisms: folate sequestration starving bone marrow precursors; LNP-induced phospholipid dysregulation; indoleamine 2,3-dioxygenase-mediated tryptophan catabolism creating immunosuppression; hepcidin-driven iron sequestration with compensatory overload; and heightened hepatic NADPH demand diverting stromal support [106].

## MATERIALS AND METHODS

### Literature search

A systematic literature search was conducted using PubMed, Scopus, and Google Scholar databases, covering the period from December 2020 to October 2025. Additional relevant studies were identified through manual screening of references in pertinent articles. The search focused on identifying studies related to haematopoietic malignancies and lymphoproliferative disorders, including leukaemias and lymphomas, as well as solid tumours potentially associated temporally or mechanistically with COVID-19 genetic vaccines. Search terms included combinations of the following keywords and MeSH terms:

“COVID-19 vaccination”, “mRNA vaccine”, “modRNA”, “cancer”, “tumour”, “malignancy”, “carcinogenesis”, “haematopoietic cancer”, “haematologic malignancies”, “leukemia”, “lymphoma”, “NK-cell leukemia”, “NK-lymphoblastic lymphoma”, “acute lymphoblastic leukemia”, “lymphoproliferative disorders”, “myeloproliferative disorders”, “side effects”, “adverse reactions”, and “vaccine safety”. Boolean operators (“AND”, “OR”) were applied to refine and combine search terms appropriately.

### Inclusion criteria and mechanistic evaluation

Eligible studies included case reports, case series, observational studies, letters to the editor, official documents from Regulatory Agencies (such as EMA, FDA, etc.), systematic reviews, and meta-analyses describing confirmed haematologic malignancies or solid tumours temporally linked or potentially related to COVID-19 vaccination, limited to English-language publications. Studies lacking confirmed diagnostic details or relevant clinical information were excluded. Mechanistic insights into the potential carcinogenic effects of COVID-19 genetic vaccines were obtained through a critical appraisal of existing molecular and immunological literature; no new experimental data were generated.

## CONCLUSIONS

The development and widespread use of modRNA vaccines have raised significant concerns globally, leading to adverse events and complications in both healthy individuals and those with pre-existing conditions. Reports of increased cases of a variety of cancers, including highly aggressive cancers, and the unexpected recurrence of cancers after decades of remission, have been independently noted by oncology experts and researchers worldwide, with several publications supporting these observations [22, 28, 35, 55–60, 62–70, 72, 107]. Although regulated as vaccines, anti-COVID modRNA vaccines also meet the definition of GTPs (Gene Therapy Products), which have been associated with tumour induction [6]. Understanding the mechanisms behind the carcinogenic effects of the modRNA COVID-19 vaccines is crucial. Immune system alterations, IgG4 class switching and notably T-cell suppression, the decreased production of IFN type I, interference with oncosuppressor genes and proteins, also through potential molecular mimicry mechanisms, inhibition of DNA repair mechanisms, inhibition of apoptosis and overexpression of cell death proteins in T-cells, are key factors facilitating neoplastic/oncogenic transformation [78–80]. Increased TGF-β production, promoting EMT, may explain the aggressive nature of observed tumours [81]. Additionally, the detection of hazardous and unexplained contamination of the modRNA vaccines with plasmid DNA sequences deriving from the manufacturing process needs to be investigated. What is the purpose of the addition of a mammalian promoter and nuclear targeting sequence from the SV40 oncovirus in the plasmid used in the manufacturing process of the Pfizer/BioNTech genetic vaccine, supposedly meant to only be used to grow copies in bacteria, where a mammalian promoter and obviously a nuclear targeting sequence are not needed? A possible reason for including the SV40 promoter sequence is that it enhances transfection efficiency and gene expression [108–110]. However, it is also capable of facilitating nuclear localization of DNA, thereby facilitating its potential integration into the genome [108–110]. Such very concerning issues must be appropriately addressed by global safety and regulatory agencies. Just as the risk of developing myocarditis and pericarditis following modRNA COVID-19 vaccination has been acknowledged [111], similar attention should be paid to assess the potential risk of developing cancer associated with the genetic vaccines. In fact, the study conducted in the research facility at the FDA White Oak campus has acknowledged that modRNA COVID-19 vaccines contain DNA contamination far exceeding the established safety limits, raising concerns about the implications for public health [85]. Since the development of COVID-19 vaccines, the modRNA technology has been quickly expanding for other diseases, and this platform is now being considered as a potential replacement for the traditional vaccine methods currently used in childhood immunizations. The potential carcinogenic effects analysed in this manuscript are specific to COVID-19 mRNA vaccines, as the literature reviewed focuses on these pharmaceutical products. However, the broader issue of double-stranded DNA contamination and its possible integration into the host genome extends beyond COVID-19 vaccines and applies to all mRNA vaccines and gene therapies. This is supported by regulatory guidelines, such as the FDA’s guidance, which can be also applied to contaminating plasmids found in mRNA vaccine production. These guidelines highlight the theoretical risk of tumorigenesis through insertional mutagenesis if DNA fragments integrate into critical genomic regions. Despite these known risks, to the authors’ knowledge, no specific integration studies have been conducted for COVID-19 mRNA vaccines, even though some independent analyses report plasmid DNA contamination levels exceeding safety thresholds. Given the expanding use of mRNA technology, thorough preclinical safety assessments, including integration studies, are urgently needed to ensure vaccine safety and public health. The carcinogenic risk associated with these technologies, which has long been known within the gene therapy field, represents an area of research that cannot be ignored, given the fundamental principle of medicine “*primum non nocere*” (first, do no harm). It is therefore crucial to perform extensive pharmacodynamic, pharmacokinetic, and genotoxicity evaluations, as well as population-based observational studies, in order to assess the potential carcinogenic risk posed by the genetic vaccines and to understand their pathogenic mechanism.
